# Characterization of Biochars Produced by Co-Pyrolysis of Hami Melon (Cantaloupes) Straw Mixed with Polypropylene and Their Adsorption Properties of Cadmium

**DOI:** 10.3390/ijerph182111413

**Published:** 2021-10-29

**Authors:** Changheng Li, Qing Huang, Haixiang Zhang, Qingqing Wang, Rixin Xue, Genmao Guo, Jie Hu, Tinghang Li, Junfeng Wang, Shan Hu

**Affiliations:** 1 College of Ecology and Environment, Hainan University, Haikou 570228, China; lichangheng16@163.com (C.L.); wangqingqing199301@163.com (Q.W.); xuerx20@yeah.net (R.X.); guogenmao123@163.com (G.G.); hj182860@163.com (J.H.); litinghang2000@163.com (T.L.); drjunfengwang2010@163.com (J.W.); hushan2000@126.com (S.H.); 2 Key Laboratory of Agro-Forestry Environmental Processes and Ecological Regulation of Hainan Province, Hainan University, Haikou 570228, China; 3 Center for Eco-Environmental Restoration Engineering of Hainan Province, Haikou 570228, China; 4 State Key Laboratory of Marine Resource Utilization in South China Sea, Hainan University, Haikou 570228, China; 5 Key Laboratory for Environmental Toxicology of Haikou, Hainan University, Haikou 570228, China; 6 College of Tropical Crops, Hainan University, Haikou 570228, China; zhanghaixiang1991@163.com

**Keywords:** Hami melon straw, polypropylene, co-pyrolysis, biochar, Cd^2+^

## Abstract

Reuse of waste from Hami melon (cantaloupes) straws (HS) mingled with polypropylene (PP) ropes is necessary and beneficial to mitigate environmental pollution. The objective of this study was to investigate the characteristics and mechanisms of Cd^2+^ adsorption on biochars produced by co-pyrolysis of HS-PP with various mixing ratios. N_2_-sorption, scanning electron microscopy (SEM), energy dispersive X-ray spectrometer (EDS), elemental analysis, Fourier-transform infrared spectroscopy (FTIR), X-ray diffraction (XRD), thermal gravity, and differential thermal gravity (TG/DTG) were applied to evaluate the physicochemical properties of materials. Batch adsorption experiments were carried out for investigating the effects of initial pH, Cd^2+^ concentration, and adsorption time. It was found that the Langmuir and pseudo-second-order models fitted best for the experimental data, indicating the dominant adsorption of co-pyrolysis biochars is via monolayer adsorption. Biochar derived at 4/1 mixing ratio of HS/PP by weight percentage had the highest adsorption capacity of 108.91 mg·g^−1^. Based on adsorption isotherm and kinetic analysis in combined with EDS, FTIR, and XRD analysis, it was concluded that the main adsorption mechanism of co-pyrolysis biochar involved the surface adsorption, cation exchange, complexation of Cd^2+^ with surface functional groups, and chemical precipitation. This study also demonstrates that agricultural wastes to biochar is a sustainable way to circular economy.

## 1. Introduction

China is a large agricultural country; accompanied with agricultural production, the generation of solid waste continues to grow rapidly. The agricultural waste production increased with the human demand for food and other agricultural products. According to the China Statistical Yearbook, the yields of crop straw are over 750 million tons every year in China. Extensive studies have been conducted on agricultural waste management worldwide, but the total utilization rate of agricultural waste in most villages of the world is less than 30% due to poor economic returns and low environmental awareness, which is a waste of these resources [1].

As one kind of synthetic polymer materials, plastics are widely used in daily life, packaging, pharmaceutical, agriculture and electronics manufacturing industries due to their low cost, light weight, and durability [2,3]. The worldwide plastic production reached 359 million tons in 2018. China is the largest plastic producer, manufacturing approximately one third of annual global plastic [4]. Due to improper disposal, 25% of the annual produced plastic ends up in the environment, making plastic an omnipresent environmental pollutant [5]. As a result, the eco-friendly utilization of these solid wastes is considered a significant challenge.

Biochar is derived from the pyrolysis of organic materials under oxygen-limited conditions. Due to its potential application for agronomic and environmental benefits, biochar has gained worldwide attention [6]. The application of biochar can improve soil physicochemical characteristics and the microbial community [7,8,9] and reduce the mobility of heavy metals and organic pollutants [10]. Biochar can be used as an economical and practical adsorbent. Due to its excellent performance in adsorption and immobilization, biochar has been considered one of the most cost-effective materials for removing heavy metals from wastewater [11].

Pyrolysis is one of the most important methods for the treatment of wastes. In recent years, biomass and plastic co-pyrolysis has attracted much attention. Co-pyrolysis of biomass and plastics can convert them into syngas, bio-oil, and biochar [12]. Most previous research about co-pyrolysis focused on the product distribution and gas and liquid product yields and characteristics. For example, it was reported that the co-pyrolysis of pine sawdust and polyethylene increased the yield and the contents of C and H, reduced the O content, and improved the quality of oil [13]. The co-pyrolysis of pine wood powder and polypropylene resulted in increased gas yield and enhanced H_2_ and CO mole fraction but reduced hydrocarbons and CO_2_ mole fraction, suggesting the improved syngas quality [14]. For comparison with pyrolysis of the only-pine-wood powder, the yield and calorific value of the bio-oil from co-pyrolysis of pine wood powder and polystyrene were apparently increased with the polystyrene ratio in feedstock [15]. The co-pyrolysis of sugarcane bagasse and high-density polyethylene not only significantly increased the yield and hydrocarbons content of bio-oil but also reduced the content of acidic compounds in bio-oil, which increased the calorific value of bio-oil and improved its quality and stability [16]. However, few research studies on the physicochemical properties of co-pyrolysis biochar are reported.

Cadmium (Cd) is an extremely toxic heavy metal, and it also has the characteristics of high mobility and easy accumulation [17]. Cd can damage human organs, including liver damage, lung damage, renal dysfunction, and osteal disorders [18]. It also has carcinogenic effects [19]. Therefore, it is critical to remove Cd^2+^ from wastewater. Hami melon (cantaloupes) is a specialty fruit of China that is sought after by consumers for its sweet flavor [20]. Hainan Province is one of the important production areas for Hami melons; the production in 2016, 2017, 2018, and 2019 was 328,800, 340,400, 402,300, and 556,600 tons, respectively. With the increasing output of Hami melon, the Hami melon straw is also increasing and thus becoming a source of pollution. In addition, polypropylene plastic ropes are used to fix the vines during Hami melon planting. After harvesting Hami melons, spare Hami melon straws (HS) and polypropylene (PP) ropes blends are commonly burned in open air or discarded in the fields, resulting in either air pollution or environmental problems. Therefore, using the HS and PP as raw materials to prepare co-pyrolysis biochar will not only reduce environmental pollution but also has potential use as adsorbent for the removal of heavy metals from wastewater since high-performance adsorbents have always been a research hot topic; additionally, no study has characterized the Cd^2+^ adsorption performance onto biochar produced from co-pyrolysis of HS and plastic.

In this study, biochars were produced by co-pyrolysis of HS and PP with various mixing ratios under anaerobic conditions at 500 °C. The prepared biochars were characterized and evaluated for Cd^2+^ removal under various conditions. Further, their adsorption properties, including the adsorption isotherms and kinetics and the adsorption mechanisms were investigated. This study can provide a theoretical reference for the resource utilization of agricultural wastes.

## 2. Materials and Methods

### 2.1. Materials

Hami melon straws (HS) and polypropylene (PP) ropes blends were collected from Wugao Village (19°45′ N, 108°87′ E), Changjiang County, Hainan Province. All chemicals used in the experiments were of analytical grade, and cadmium nitrate (Cd(NO_3_)_2_·4H_2_O), sodium nitrate (NaNO_3_), nitric acid (HNO_3_), and sodium hydroxide (NaOH) were purchased from Aladdin Biochemical Technology Co., Ltd., Shanghai, China. Deionized water was used in all the experiments.

### 2.2. Biochar Preparation

The HS and PP were washed with distilled water several times and placed in an oven at 80 °C until reaching constant weight. Then, raw materials were pulverized and passed through a 30-mesh sieve for subsequent use. The batches of HS-PP were prepared under the PP blending ratio of 0, 10%, 20%, and 30% (W/W). Approximately 100 g of feedstock was tightly placed in a 300 L ceramic pot and then pyrolyzed in a muffle furnace at the peak temperature 500 °C for 3 h under oxygen-limited conditions with a heating rate of 5 °C·min^−1^. The obtained biochars were referred to as BC0, BC10, BC20, and BC30, respectively, according to the PP content in blends. All samples were stored in brown bottles for further use.

### 2.3. Characterization of Biochars

The yield of biochar was calculated as a percentage ratio of the weight of char after pyrolysis and feedstock prior to pyrolysis. Ash content of biochar was measured by heating it at 800 °C for 4 h in a muffle furnace. The biochar was mixed with deionized water at the ratio of 1:20 (*w*/*v*), stirred for 2 h, and then measured the pH value with a pH meter. The C, N, H, and O contents of biochar were measured by an elemental analyzer (Vario Microcube, Elementar, Langenselbold, Germany). The specific surface area of biochar was determined by the Brunauer–Emmett–Teller (BET) method using a gas sorption analyzer (ASAP2060, Micromeritics, Norcross, GA, USA). The morphology and structure of biochar were analyzed using a scanning electron microscope (SEM) (Verios G4 UC, Thermo Fisher Scientic, Waltham, MA, USA). The energy dispersive X-ray spectrometer (EDS) was used to analyze the surface element of biochars. Fourier-transform infrared (FTIR) spectroscopy was used to identify the surface functional groups of biochars (IS50, Thermo Fisher Scientic, USA). Crystalline constituents of biochars were determined by X-ray diffraction (XRD) (D/MAX2500, Rigaku, Tokyo, Japan). Volatile matter content and the weight loss from 100 °C to 850 °C of different materials were measured by using a thermal gravimetric analyzer (TGA8000, Perkin-Elmer, Waltham, MA, USA).

### 2.4. Adsorption Experiment

#### 2.4.1. Sorption of Cd^2+^

Stock solution of 1000 mg·L^−1^ Cd^2+^ was prepared by dissolving Cd(NO_3_)_2_·4H_2_O in 0.01 mol·L^−1^ NaNO_3_, the background solution. The 0.02 g biochar was added in to 20 mL Cd^2+^ solution in polyethylene centrifuge tubes. All tubes were shaken at 300 rpm for 24 h at 25 °C in a constant temperature vibrator incubator. Afterward, the samples were collected, and the supernatant solution was filtered through 0.45-μm membrane filter. The residual concentration of Cd^2+^ was determined by atomic absorption flame spectrometer.

The adsorption capacity of different biochars were calculated according to the following equation:

(1) $$Q_{e}=\frac{\left(C_{0}{-C}_{e}\right)V}{M}$$

where *C*_0_ and *C_e_* are the concentrations (mg·L^−1^) of Cd^2+^ in the aqueous phase at the initial and the equilibrium, respectively. *M* (g) is the mass of added biochar, and *V* (L) is the volume of solution.

#### 2.4.2. Effect of Solution pH on Cd^2+^ Adsorption

The effect of pH on Cd^2+^ sorption by biochar was carried out by adjusting the 150 mg·L^−1^ Cd^2+^ solution initial pH from 2 to 8 with either 0.1 M HNO_3_ or 0.1 M NaOH. The other steps were the same as above.

#### 2.4.3. Adsorption Isotherms of Biochars for Cd^2+^

The adsorption capacity of biochars with different concentrations of Cd^2+^ was carried out by adding 0.02 g of the biochar to 50 mL polyethylene centrifuge tubes containing 20 mL Cd^2+^ solution, the initial Cd^2+^ concentration in the range of 10–400 mg·L^−1^. The centrifuge tubes were shaken using an incubator shaker at 300 rpm and 25 °C for 24 h. After that, the mixtures were collected, and supernatant solution was filtered through a 0.45-μm membrane filter for analysis using the same method.

The adsorption isotherm curves were fitted by Langmuir model and Freundlich model; the models are as follows:

Langmuir model:

(2) $$Q_{e}=\frac{Q_{m}K_{L}C_{e}}{1+K_{L}C_{e}}$$

Freundlich model:

(3) $$Q_{e}{=K}_{F}C_{e}{}^{1/n}\;$$

where *C_e_* (mg·L^−1^) is the equilibrium concentration of Cd^2+^ in the solution, *Q_e_* (mg·g^−1^) is the adsorbed amount of Cd^2+^ at adsorption equilibrium, *Q_m_* (mg·g^−1^) is the maximum sorption amount, *K_L_* (L·mg^−1^) is the Langmuir constant, and *K_F_* (mg·g^−1^) (L·mg^−1^)^1/*n*^ and 1/*n* are Freundlich constants.

#### 2.4.4. Adsorption Kinetics of Biochars for Cd^2+^

The adsorption kinetics of the biochars for Cd^2+^ were carried out by adding 0.02 g of the biochar to 50 mL polyethylene centrifuge tubes containing 20 mL of the 150 mg·L^−1^ Cd^2+^ solution. The centrifuge tubes were shaken using an incubator shaker at 300 rpm and 25 °C for different time interval (0, 0.1, 0.25, 0.5, 1, 2, 4, 7, 12, 18, and 24 h). Samples were collected, and the supernatant solution was filtered through a 0.45-μm membrane filter and then analyzed the concentration of Cd^2+^ in the filtrate.

Pseudo-first-order kinetic equation and pseudo-second-order kinetic equation were employed to model the adsorption data. Two models can be expressed as follows:

Pseudo-first-order kinetic model:

(4) $$Q_{t}{=Q}_{e}\left({1-e}^{{-k}_{1}t}\right)$$

Pseudo-second-order kinetic model:

(5) $$Q_{t}=\frac{k_{2}Q_{e}{}^{2}t}{1+k_{2}Q_{e}t}\;$$

where *Q_t_* and *Q_e_* (mg·g^−1^) represent the adsorption amount of Cd^2+^ at time *t* (h) and at equilibrium. *k*_1_ (h^−1^) and *k*_2_ (g·mg^−1^·h^−1^) are the adsorption rate constants of pseudo-first-order kinetic model and pseudo-second-order kinetic model, respectively.

In this study, the kinetics and adsorption isotherms were fitted by Origin 2018 software.

## 3. Results and Discussion

### 3.1. Basic Physicochemical Properties of Biochars

#### 3.1.1. Yield, Ash Content, pH, and Elementary Composition

The yield and ash content of biochar from co-pyrolysis of HS and PP are influenced by mixing ratios (Table 1). The biochar yield decreased by 30.27% when PP contents in feedstock increased from 0 to 30%. Ash was also decreased when the PP was added: the ash content decreased from 40.49% at 0 to 38.98% at 30%. These results are consistent with the results of activated carbon produced by co-pyrolysis of sycamore sawdust and PP [21]. The values of pH were higher than 11 for all the biochars due to the decomposition of acidic functional groups in feedstock and the accumulation of inorganics during the pyrolysis. However, the addition of PP showed no significant effects on the pH values of the biochars.

The results of elemental analysis revealed that, compared with the HS, C content in biochars increased, whereas contents of O and H decreased, suggesting that part of the O and H elements were lost during pyrolysis in form of water. The C content in biochar varied from 36.90% to 40.50%; the C content decreased first and then increased with the increase of PP content. The lowest C content of 36.90% was observed in BC20. The content of N was in accordance with the trend of C content, but the contents of H and O increased first and then decreased. As reported, variation in the contents of C, O, N, and H might suggest changes in functional groups [11]. The ratios of H/C and O/C represent the indices of aromaticity and polarity [22]. These values increased first and then decreased with the increase of PP content. It was found that the highest ratios of H/C and O/C were obtained by BC20, which indicated that the biochar had the lowest aromaticity and the highest polarity when the PP content was 20%. This may be due to the volatiles increased with increasing of PP content, resulting in a fixed C-content decrease and ratio of H/C increase; when the PP content was 30%, considering that some volatile fraction of PP remains in the biochar, the content of C increased, and the value of H/C decreased [23]. It was reported that the significant positive correlation between the ratio of H/C and O/C and metal adsorption amounts for biochars [24].

#### 3.1.2. Surface Area and Pore Analysis

The surface area and pore structure of biochar played an important role in the adsorption process of heavy metals, and the diffusion rates for Cd^2+^ is inconsistent because of the presence of different pore structures [25]. The specific surface area, average pore diameters, and pore volumes of biochars are shown in Table 2. The BET surface area was obviously increased when the PP was added. When the PP content was 20%, the specific surface area reached the maximum of 12.58 m^2^·g^−1^. Higher specific surface area was favorable for the adsorption of heavy metals by biochar. The total pore volume of biochars also increased first and then decreased with the increases of PP content. The highest total pore volume was obtained by BC20. This may be due to the fact that PP is rich in volatiles, and the thermal degradation of PP occurs in the range of 410–500 °C [26]. When HS was mixed with PP, the PP was completely degraded at approximately 500 °C, the original space occupied by the PP formed a pore structure. At the same time, porous effect enhanced heat transfer effect, causing the pyrolysis process more homogeneous as a result [21]; in addition, the synergistic effects during the co-pyrolysis of biomass and plastic are also beneficial for the degradation of feedstock [27]. When the PP blending was more than 20%, the original pore structure was destroyed because the material is ablated during the process, resulting in decrease of specific surface area and pore volume. Table 2 displays the average pore diameter decrease when the PP was added. It was found that the highest specific surface area and the total pore volume were obtained by BC20, indicating that the generation of pore structure was promoted by the interaction between HS and PP when the percentage of PP was 20%.

#### 3.1.3. Thermogravimetric Analysis of Raw Materials

**Figure 1:**
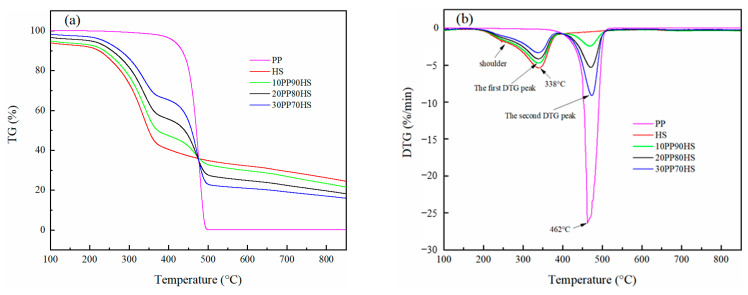
(**a**) TG and (**b**) DTG curves of HS, PP, and their blends pyrolyzed at 10 °C·min^−1^ (the number preceding the abbreviated letters represents the material content in blends).

The thermogravimetry (TG) and differential thermogravimetry (DTG) curves of HS, PP, and their mixtures are shown in Figure 1. HS and PP had different pyrolytic behavior because of their differences of molecular structure. HS was mainly composed of cellulose, hemicellulose, and lignin, and PP was a linear polymer [26]. For comparison with PP, HS exhibited a lower initial pyrolysis temperature and a broader degradation range. The degradation of HS mostly occurred at 200–550 °C, which was composed of two stages. The fast weight-loss stage took place from 200 to 400 °C, with the maximum loss rate of 5.40%·min^−1^ at 338 °C, which was consistent with previous research [28]. There was a shoulder on the left of the single peak, indicating that the decomposition process overlapped in this district. It was reported that the degradation of hemicellulose and cellulose mostly occur at 190–380 °C and 250–380 °C, respectively [29]. The degradation of lignin takes place from 150 to 900 °C, which is a wide range and without observable DTG peak [30]. Therefore, the peak at 338 °C corresponded to the decomposition of cellulose and lignin, and the shoulder represented the decomposition of hemicellulose and lignin. A slow loss of mass stage with a broad range of temperatures above 400 °C was caused by the lignin degradation [2]. The yield of solid residue from HS was 24.40% at 850 °C. The degradation behavior of PP was reported in a previous work [31], which showed a one-step process with only one peak. Compared to HS, PP degradation mainly occurred from 410 to 500 °C with a narrow temperature range. In temperature section, the maximum loss rate for PP was 26.41%·min^−1^ at 462 °C, and PP degraded completely at the final temperature with almost no residual char because of its high volatile content [31].

When the HS and PP were mixed, the weight-loss curve of the mixture lays between the ones of the individual HS and PP. The co-pyrolysis process of HS-PP blends occurred in two stages. The first stage was mainly associated with the HS decomposition, and the second stage was responsible for the degradation of PP. As illustrated in Figure 1, as the PP ratio increased, the intensity of the first DTG peak decreased, whereas the intensity of the second DTG peak increased. Compared with HS, the initial pyrolysis temperature (temperature for initial mass loss) of the mixture in the first stage was higher than that of individual HS, and the temperature at the maximum weight-loss rate for the blends was almost higher than that of individual HS. This was due to the PP softening, resulting in the coating of the biomass particles and thereby inhibiting the release of volatile species [32]. In addition, compared with individual PP, the terminated pyrolysis temperature (temperature for final mass loss) of all blends at the second stage were 516.96 °C, 518.77 °C, and 519.23 °C, respectively. It can be observed that this temperature of the blends was reduced around 0.99–3.26 °C compared with the individual PP (520.22 °C), which indicated that the addition of PP promotes the pyrolysis of the mixture. The decomposition characteristic temperature reflected that a synergistic effect might exist during the co-pyrolysis of HS and PP [31].

#### 3.1.4. Surface Morphology

**Figure 2:**
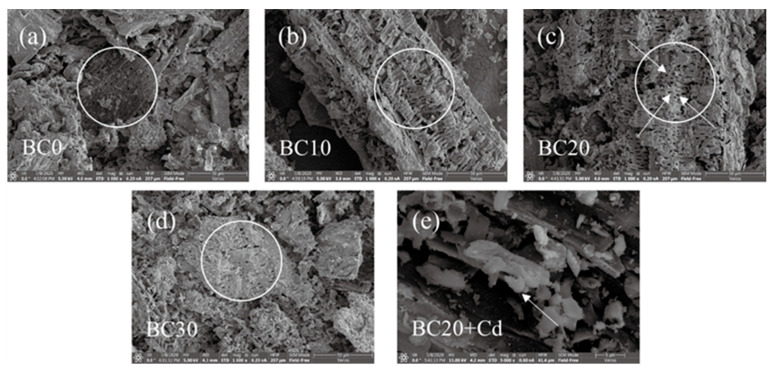
SEM images of (**a**) BC0, (**b**) BC10, (**c**) BC20, (**d**) BC30, and (**e**) BC20 after Cd^2+^ adsorption.

The SEM images of biochars are shown in Figure 2. It can be found that PP affects the morphology of biochar. There was fewer porous structure on the surface of biochar prepared without the addition of PP, while the surface of the biochar was rough, and more pores were observed when the PP was added. It was clear that better developed pore structure was observed in the biochar when the amount of PP added in blends was 20%.

#### 3.1.5. FTIR Analysis

**Figure 3:**
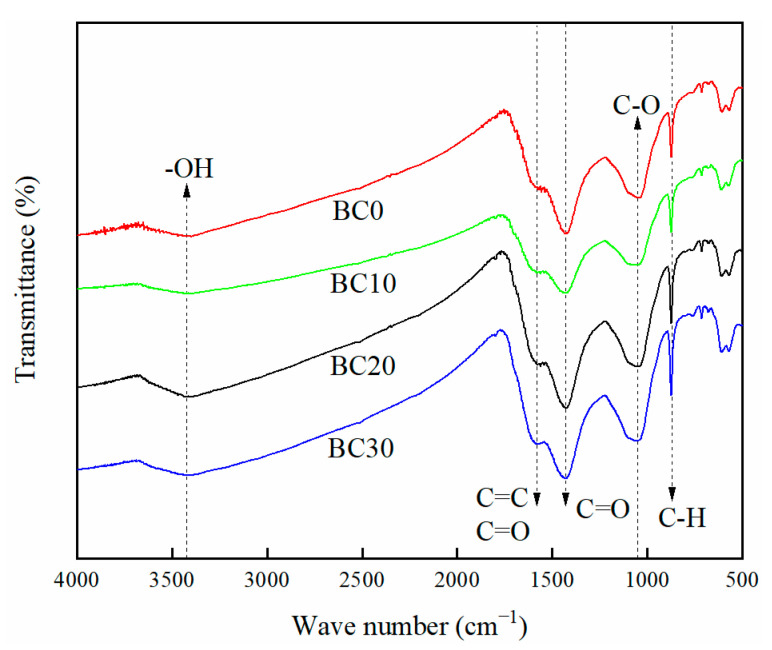
Comparison of FTIR spectra plotted for biochars derived from HS-PP blends.

The functional groups on the surface of biochar play an important role in the adsorption process of heavy metals. FTIR spectroscopy was introduced to identify the surface functional groups, and the FTIR spectra of BC0, BC10, BC20, and BC30 are presented in Figure 3. The peak near 3421 cm^−1^ of all biochars were ascribed to the O-H stretching vibration [33]. The intensity of this peak increased first and then decreased with the increase of PP content in feedstock; this result was consistent with the change of O content observed in elementary composition. The shoulder near 1580 cm^−1^ corresponded to the C=O, C=C stretching vibration [34]. The peaks at 1429 cm^−1^ and 1048 cm^−1^ were assigned to C=O stretching vibration in CO_3_^2−^ and C-O stretching vibration, respectively [35]. The peak at 873 cm^−1^ was attributed the aromatic C-H bending vibration [36], and the peak was more apparent in BC30 than in other biochars, indicating that more aromatic structure were preserved. Even though there was a variation in the intensities of the biochars’ function groups, there was no observable differences in their types with the change in proportion of PP in feedstock.

#### 3.1.6. XRD Analysis

**Figure 4:**
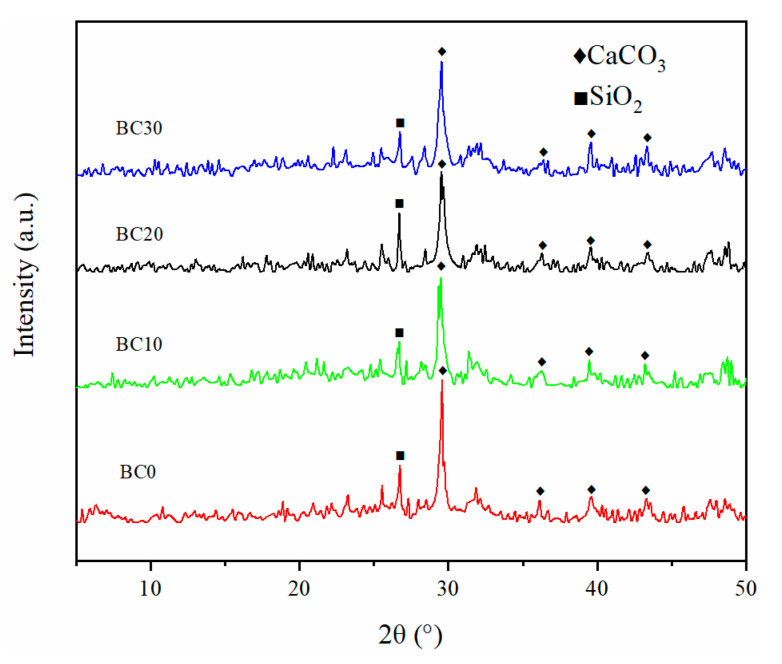
Comparison of XRD patterns plotted for biochars derived from HS-PP blends.

To understand the mineral crystals existing in biochar, XRD analysis was performed on these simples. The XRD patterns of BC0, BC10, BC20, and BC30 are shown in Figure 4. The sharp peak at 26.7° corresponded to the characteristic peak of SiO_2_ [37]. In addition, diffraction peaks at 29.5°, 36.3°, 39.6°, and 43.4° indicated the presence of CaCO_3_ [11], which were observed in all samples. These results suggested that the prepared biochars had similar crystal species.

### 3.2. Adsorption Performance in Solution

#### 3.2.1. Effect of Solution pH on Cd^2+^ Adsorption

**Figure 5:**
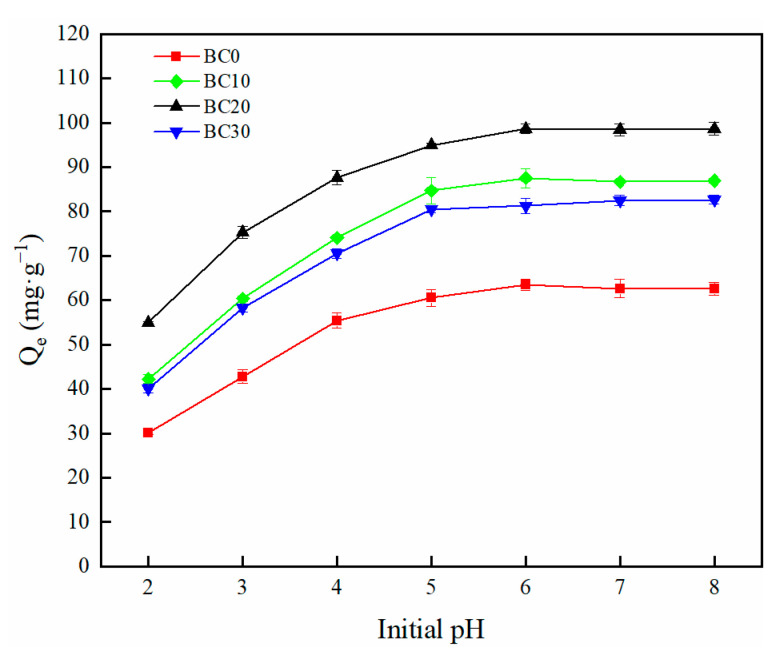
The effect of pH (2–8) on the adsorption of Cd^2+^ by different biochars.

The initial pH of the solution is one of the most important factors that determine the adsorption process of pollutant [38]. The effect of initial solution pH in the range from 2 to 8 on Cd^2+^ adsorption by biochars is shown in Figure 5. The adsorption capacity of biochar for Cd^2+^ gradually increased with the increasing initial solution pH. The adsorption capacity of biochar increased rapidly when the pH increased from 2 to 4. Thereafter, the adsorption capacity of Cd^2+^ by biochar increased slowly and tends to be stable when the increase in initial pH ranged from 4 to 6. When the pH of the solution increased from 2 to 6, the adsorption capacity increased from 30.14 mg·g^−1^ to 63.47 mg·g^−1^ for BC0, from 42.21 mg·g^−1^ to 87.53 mg·g^−1^ for BC10, from 54.99 mg·g^−1^ to 98.68 mg·g^−1^ for BC20, from 40.10 mg·g^−1^ to 81.25 mg·g^−1^ for BC30. The adsorption capacity of biochar maintained stable and had no obvious difference with the initial pH from 6 to 8. These results are in line with previous studies [24,39]. Figure 5 demonstrates the low Cd^2+^ adsorption onto biochar at low solution pH. The possible reason for this phenomenon is that the functional groups (e.g., C-O, C=O) on the surface of the biochar were protonated and led to electrostatic repulsion to the Cd^2+^ in solution and H^+^ in the solution, which may compete with Cd^2+^ for adsorption sites and thus affected the adsorption performance of Cd^2+^ [40]. When the solution pH increases, the concentration of H^+^ in the solution decreased, and the oxygen-containing functional groups (e.g., -OH, -COOH) were deprotonated, resulting in the surface of biochar becoming more negative and Cd^2+^ adsorption by biochar increasing through electrostatic interaction. In addition, the formation of Cd^2+^ precipitate Cd(OH)_2_ at higher pH was also an important factor for the Cd^2+^ adsorption [25]. Therefore, the conditions from weak acid to neutral were favorable for Cd^2+^ adsorption. In addition, at a constant pH, BC20 had the highest capacity for Cd^2+^ adsorption among the biochars. In this study, the optimum initial pH of the solution was 6.

#### 3.2.2. Adsorption Isotherms

**Figure 6:**
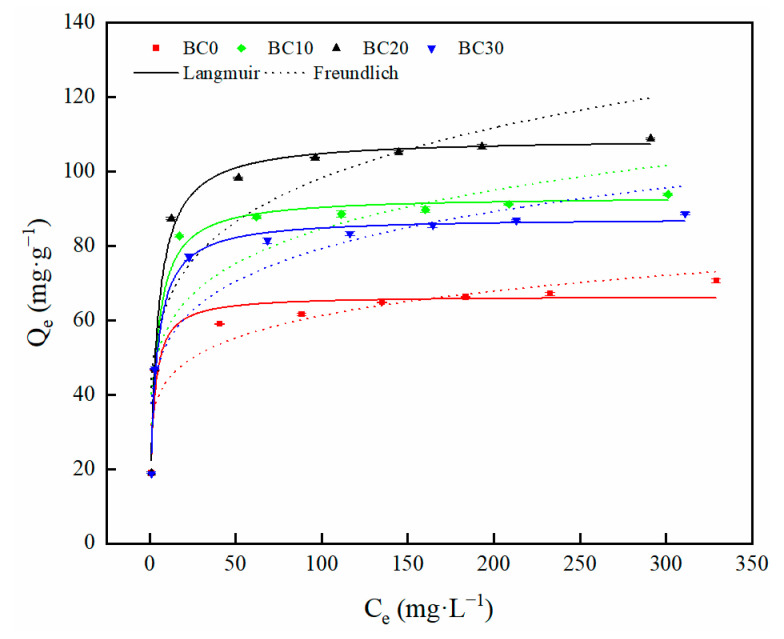
Adsorption isotherms of Cd^2+^ by biochars: Langmuir and Freundlich models.

The adsorption isotherm of Cd^2+^ by biochar are illustrated in Figure 6. As the initial Cd^2+^ concentration increased, the adsorption capacity of biochars gradually increased and the trend slowed down. By increasing the initial concentration, the Cd^2+^ equilibrium adsorption capacity of BC20 was higher than other biochars, which could possibly be due to the microstructure (Table 2) and surface functional groups (Figure 3). From the SEM images of biochars (Figure 2), BC20 exhibited a more porous structure, suggesting that a good porous structure may be beneficial to Cd^2+^ removal.

When the initial concentration of Cd^2+^ was 400 mg·L^−1^, the maximum adsorption capacities for BC0, BC10, BC20, and BC30 were 70.66, 93.83, 108.87, and 88.71 mg·g^−1^, respectively. As the most frequently used models to describe the sorption characteristics, the Langmuir and Freundlich models are considered suitable for monolayer and heterogeneous multilayer adsorption, respectively [41]. The corresponding parameters of isotherm models are shown in Table 3. Comparisons of these data revealed that the fitting isotherms did not follow the Freundlich model appropriately. The experimental data for BC0, BC10, BC20, and BC30 correlated quite well with the Langmuir model, as the fitting correlation coefficient values (*R*^2^) were greater than those of the Freundlich model. This also indicated that the Cd^2+^ adsorption process by biochar is a monolayer adsorption [42]. It was found that the experimentally determined maximum adsorption capacity was close to the predicted maximum adsorption capacity estimated using the Langmuir model.

BC20 had the strongest adsorption capacity among all biochars. According to the Langmuir model, *R_L_* (*R_L_* = 1/(1 + *K_L_* × *C*_0_)) is a dimensionless constant separation factor that can be used to indicate the type of the isotherm [43]. 0 < *R_L_* < 1 indicates the adsorption process is favorable; *R_L_* = 1 indicates linear; *R_L_* > 1 indicates unfavorable; *R_L_* = 0 indicates irreversible [44]. In this study, the *R_L_* values for all biochars were in the range of 0 to 1, indicating that adsorption of Cd^2+^ by all biochars were favorable process. In addition, the 1/*n* value obtained from the Freundlich model indicates the adsorption favorability. It is generally considered that a value range from 0.1 to 0.5 demonstrates easy adsorption [45]. The 1/*n* values of Cd^2+^ adsorption onto BC0, BC10, BC20, and BC30 were 0.148, 0.168, 0.185, and 0.170, respectively, suggesting a favorable adsorption process due to the fact that the factors were less than 0.5.

#### 3.2.3. Adsorption Kinetics

**Figure 7:**
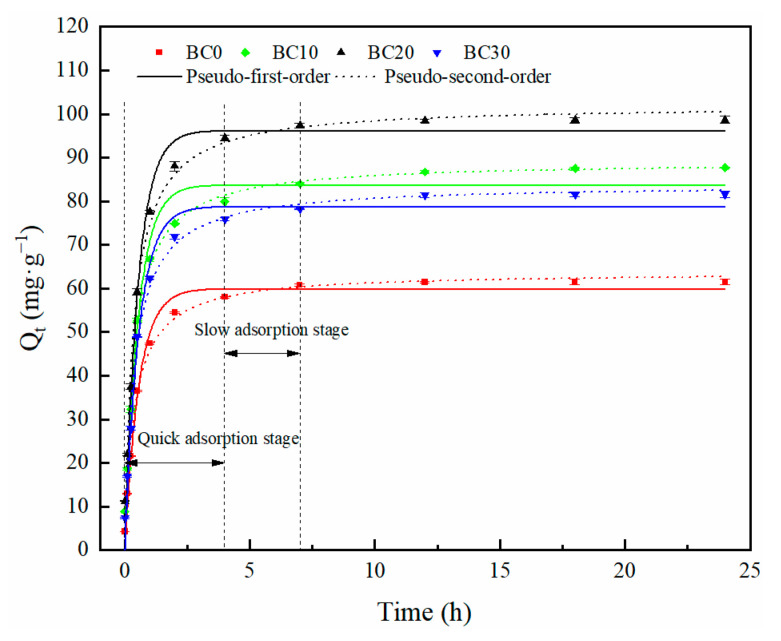
Adsorption kinetics of Cd^2+^ onto biochars: pseudo-first-order model and pseudo-second-order model.

Examining adsorption kinetic can be helpful for understanding the adsorption process. The experimental data for the Cd^2+^ adsorption kinetics of different biochars are displayed in Figure 7. The adsorption of Cd^2+^ by biochar includes two processes: a rapid adsorption process where a large amount of Cd^2+^ was adsorbed in the first 4 h, and then a slow adsorption process until the equilibrium of adsorption. The Cd^2+^ adsorbed on these biochars all reached equilibrium within 7 h. The adsorption primarily occurred on the outer surfaces of biochar at quick adsorption stage; its adsorption mechanism was expected to be related to both chemical and physical adsorption. At the next stage, Cd^2+^ entered into the pores of the biochar and combined with active sites, and the adsorption process was slow until it reached the equilibrium [39]. Compared with other biochars, the equilibrium amounts adsorbed by BC20 were higher (Figure 7). This result was consistent with the phenomenon observed in SEM images (Figure 2), indicating that more pores were beneficial to Cd^2+^ removal.

The Cd^2+^ equilibrium adsorption capacity for BC0, BC10, BC20, and BC30 was 61.56, 87.73, 98.71, and 81.60 mg·g^−1^, respectively, and the Cd^2+^ equilibrium adsorption capacity of BC20 was the highest among all biochars. Experimental data were fitted by pseudo-first-order and pseudo-second-order kinetics models to analyze adsorption kinetics mechanism. The two kinetic models fitting parameters are exhibited in Table 4. The *R*^2^ of the four biochars for pseudo-second-order model were all higher than those of the pseudo-first-order model, which indicated that the adsorption of Cd^2+^ followed the pseudo-second-order better than it followed the pseudo-first-order one. As shown in Table 4, the adsorption capacity obtained by fitting with the pseudo-second-order model of all biochars was 63.73, 89.11, 102.03, and 83.83 mg·g^−1^, respectively. These masses were close to the experimental sorption value. Therefore, the Cd^2+^ adsorption mechanism followed the pseudo-second-order kinetic model, and it was inferred that chemical adsorption is the rate-limiting step [46]. Similar results have been reported for sugarcane, neem, and plastic biochar for the adsorption of heavy metals [38].

### 3.3. Adsorption Mechanism of Cd^2+^ by Biochars

#### 3.3.1. Surface Adsorption

Multiple mechanisms are involved in heavy metal adsorption onto biochar. From the findings of the physicochemical characteristics and the Cd^2+^ adsorption behaviors of the biochars, the adsorption mechanisms of these biochars for Cd^2+^ mainly include physical adsorption and chemical adsorption. The specific surface area, total pore volume, and average pore diameter of the biochars are presented in Table 2. The average pore diameters were 21.82 nm, 15.71 nm, 18.11 nm, and 16.77 nm for BC0, BC10, BC20, and BC30, respectively, suggesting that these biochars belong to mesoporous materials and are suitable for adsorbents. Among the biochars, BC20 had the highest specific surface area and the biggest total pore volume. Its specific surface area is 1.82 times as much as BC0, and its total pore volume is 1.59 times as large as BC0. The adsorption results show that BC20 displayed best adsorption performance among the four biochars, indicating that the larger specific surface area and pore volume were beneficial to heavy metals removal. In summary, surface adsorption is one of the mechanisms of co-pyrolysis biochar to adsorb Cd^2+^.

#### 3.3.2. Ion Exchange

**Figure 8:**
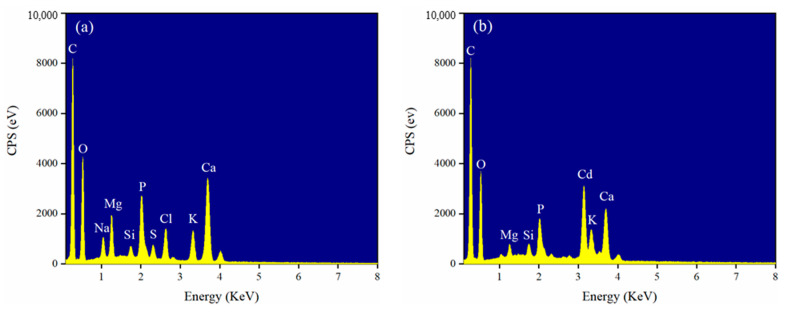
EDS spectra of (**a**) pristine BC20 and (**b**) BC20 after Cd^2+^ adsorption.

Ion exchange play an important role in the heavy metals sorption process. It was reported that K^+^, Ca^2+^, Na^+^, and Mg^2+^ in rice straw biochar could be replaced by Cd^2+^ during the adsorption process, and the adsorption amount of Cd^2+^ caused by the cation exchange reaction accounts for 56.14% of the total adsorption [47]. As observed in Figure 8, the peak of Cd was detected after adsorption, and the peaks represent K, Ca, Na, and Mg decreasing or even disappearing after Cd^2+^ adsorption, indicating that these cations on the biochars contribute to Cd^2+^ adsorption through cation exchange. A similar result was found in the adsorption of Cd^2+^ by water hyacinth stems biochar [19].

#### 3.3.3. Complexation with Surface Functional Groups

**Figure 9:**
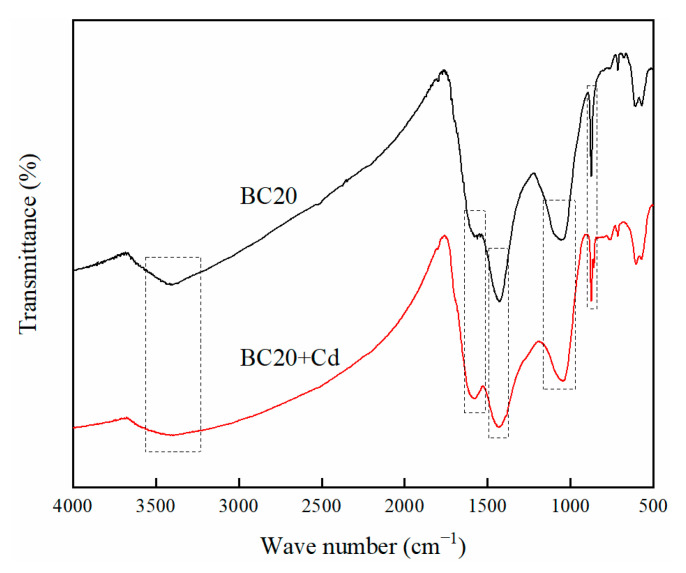
FTIR patterns of BC20 before and after Cd^2+^ adsorption.

FTIR spectroscopy was performed to detect the interactions between Cd^2+^ and the surface functional groups of biochars in the adsorption process. As shown in Figure 9, the FTIR spectrum changed obviously after BC20 adsorbs Cd^2+^ compared with BC20, which indicate that some surface functional groups were involved in Cd^2+^-adsorption process. The FTIR spectroscopy of BC20 after Cd^2+^ adsorption suggest a significant reduction in the peaks of -OH and C-O stretching vibration at 3399 cm^−1^ and 1047 cm^−1^, indicating that complexation with oxygen-containing groups contribute to Cd^2+^ adsorption. These results were in line with other researchers who reported that the removal heavy metals were attributed to surface complexation with -OH and C-O groups for the edible fungus waste substrate biochar [48]. The intensity of the peak at 1600 cm^−1^ increased after adsorption, indicating that the C=C group was involved in the Cd^2+^-adsorption process, and interaction of Cd^2+^ with π electrons might be account for Cd^2+^ adsorption [49]. After BC20 adsorbed Cd^2+^, the peak intensity of C=O stretching vibration changed significantly at 1429 cm^−1^ (Figure 9), suggesting that the surface complexation of Cd^2+^ with CO_3_^2−^ plays a vital role in the adsorption process [50]. Furthermore, the intensity of the peak at 873 cm^−1^ changed significantly after Cd^2+^ adsorption, possibly because the aromatic -CH group provide π electrons during the Cd^2+^ adsorption [46].

#### 3.3.4. Chemical Precipitation

**Figure 10:**
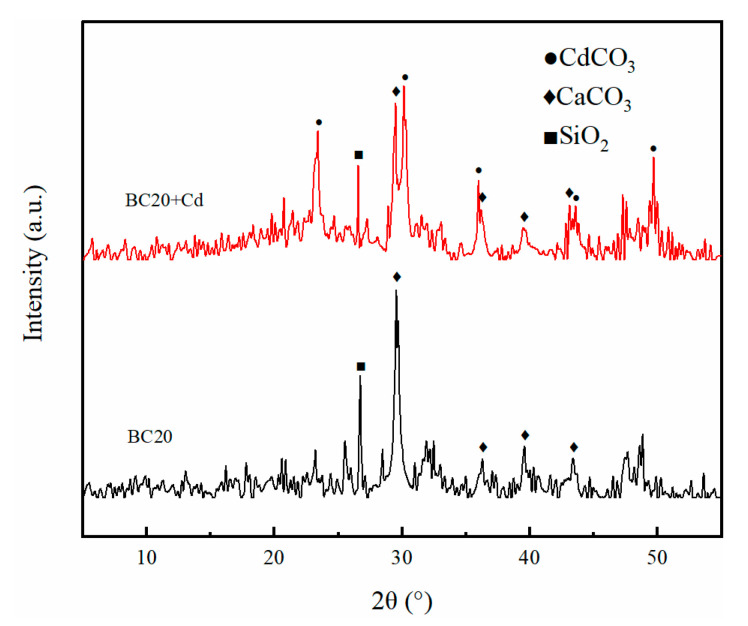
XRD patterns of BC20 before and after Cd^2+^ adsorption.

Some anions (e.g., CO_3_^2−^, PO_4_^3−^, OH^−^) released from biochars can precipitate with Cd^2+^, and this has been reported as an important adsorption mechanism for biochar [51]. Relative to the original biochar, a large number of white granular crystals dispersedly scattered on the surface of BC20 after Cd^2+^ adsorption (Figure 2). To identify the crystalline mineral phases for Cd^2+^ adsorption, BC20 was comparatively analyzed before and after Cd^2+^ adsorption by XRD (Figure 10). The new peaks at 23.4°, 30.2°, 35.9°, 43.6°, and 49.7° after Cd^2+^ adsorption represent the characteristic peaks of CdCO_3_ [51], indicating that the white granular precipitates identified were CdCO_3_. Similar results have been reported for rice straw biochar for Cd^2+^ adsorption [47].

### 3.4. Application of Co-Pyrolysis Biochar

The adsorption capacity of biochar to heavy metals varies with different raw materials and preparation methods. Table 5 summarizes the Cd^2+^ adsorption capacity of the adsorbents in this study and those that have been published in previous studies. By comparing the adsorption performance of different materials in the Table 5, it can be found that the theoretical adsorption capacity of Cd^2+^ by biochar from co-pyrolysis of HS and PP was higher than that of other biochar. The advantage of high adsorption capacity of BC20 for the effective removal of Cd^2+^ indicates that BC20 has great application potential for water remediation. In addition, HS and PP as agricultural wastes are easily achieved and low cost.

## 4. Conclusions

The physicochemical properties and the Cd^2+^ adsorption capacity of co-pyrolytic biochars were affected by the addition of PP. When the PP content was 20%, the adsorption capacity of Cd^2+^ reached the maximum of 108.91 mg·g^−1^, indicating that BC20 can be used as an effective sorbent for removing Cd^2+^ from aqueous solutions. Among the various factors influencing the adsorption capacity of Cd^2+^ by biochars, it was found that the adsorption performance of biochars was influenced by pH, adsorption time, and concentration of Cd^2+^. The adsorption data of four biochars fitted well with Langmuir model and pseudo-second-order kinetics model. The analysis of BET and pore size distribution, SEM-EDS, XRD, and FTIR before and after adsorption of Cd^2+^ by BC20 indicated that surface physical adsorption, ion exchange reaction between Cd^2+^ and cations, Cd-π interaction, complexation reaction with oxygen-containing functional groups, and precipitation reaction of Cd^2+^ with CO_3_^2−^ were main mechanisms of adsorption. The findings of these experiments revealed that BC20 is considered as a potential adsorbent for Cd^2+^ removal from wastewater. This work also provides a new insight into the agricultural waste treatment problems.

## Figures and Tables

**Table 1:** Yield, ash contents, pH, elemental analysis, and atomic ratio of biochars derived from HS-PP blends.

Biochars	Yield (%)	Ash (%)	pH	C (%)	N (%)	O (%)	H (%)	H/C	O/C
BC0	32.64	40.49	11.54	40.50	2.17	15.48	1.36	0.0336	0.382
BC10	29.51	39.55	11.54	37.86	1.88	19.26	1.45	0.0383	0.509
BC20	26.41	39.14	11.44	36.90	1.81	20.67	1.48	0.0401	0.560
BC30	22.76	38.98	11.38	39.35	1.97	18.27	1.43	0.0363	0.464

**Table 2:** Microstructure properties of biochars derived from HS-PP blends.

Biochars	BET Surface Area (m^2^·g^−1^)	Total Pore Volume (10^−2^·cm^3^·g^−1^)	Average Pore Diameter (nm)
BC0	6.90	2.85	21.82
BC10	11.95	3.87	15.71
BC20	12.58	4.53	18.11
BC30	11.78	4.17	16.77

**Table 3:** Adsorption isotherm constants for the Cd^2+^ by biochars derived from HS-PP blends.

Biochars	Langmuir			Freundlich		
	*Q_m_* (mg·g^−1^)	*K_L_* (L·mg^−1^)	*R* ^2^	*K_F_* (mg·g^−1^) (L·mg^−1^)^1/*n*^	1/*n*	*R* ^2^
BC0	66.53	0.47	0.937	30.95	0.148	0.827
BC10	93.29	0.30	0.984	38.94	0.168	0.769
BC20	108.91	0.25	0.993	41.82	0.185	0.809
BC30	87.52	0.30	0.983	36.17	0.170	0.808

**Table 4:** Fitting parameters of the pseudo-first-order and pseudo-second-order kinetics models by biochars.

Biochars	*Q_e_*_,*exp*_^1^ (mg·g^−1^)	Pseudo-First-Order			Pseudo-Second-Order		
		*k*_1_ (h^−1^)	*Q_e_*_,*cal*_^2^ (mg·g^−1^)	*R* ^2^	*k*_2_ (g·mg^−1^·h^−1^)	*Q_e_*_,*cal*_^3^ (mg·g^−1^)	*R* ^2^
BC0	61.56	1.76	59.91	0.985	0.0395	63.73	0.991
BC10	87.73	1.86	83.68	0.972	0.0297	89.11	0.987
BC20	98.71	1.87	96.17	0.977	0.0268	102.03	0.984
BC30	81.60	1.78	78.75	0.980	0.0303	83.83	0.987

^1^ *Q_e_*_,*exp*_ is the actual adsorption capacity at equilibrium. ^2^ *Q_e_*_,*cal*_ is the equilibrium sorption capacity calculated by the pseudo-first-order model. ^3^ *Q_e_*_,*cal*_ is the equilibrium sorption capacity calculated by the pseudo-second-order model.

**Table 5:** Comparison of Cd^2+^ adsorption capacity (*Q_m_*) with other reported adsorbents.

Adsorbents	Cd^2+^ Adsorption Capacity (mg·g^−1^)	References
Mango peel biochar (500 °C)	16.01	[52]
*Lantana camara* L biochar (500 °C)	54.43	[11]
*Mikania micrantha* biochar (500 °C)	84.96	[11]
*Ipomoea cairica* biochar (500 °C)	72.46	[11]
Rice straw biochar (400 °C)	37.14	[47]
Rice straw biochar (700 °C)	65.40	[47]
Rice husk biochar (300 °C)	62.75	[51]
Rice husk biochar (500 °C)	77.37	[51]
Rice husk biochar (700 °C)	93.50	[51]
Lucerne shoot biochar (550 °C)	6.28	[53]
Peanut husk biochar (500 °C)	28.99	[54]
BC20 (500 °C)	108.91	This study

## Data Availability

Not applicable.
